# Rubric vs. numeric rating scale: agreement among evaluators on endodontic treatments performed by dental students

**DOI:** 10.1186/s12909-023-04187-3

**Published:** 2023-03-30

**Authors:** Nuria Escribano, Virginia Belliard, Bruno Baracco, Dayana Da Silva, Laura Ceballos, M. Victoria Fuentes

**Affiliations:** Faculty of Health Sciences, IDIBO Research Group, Rey Juan Carlos University, Madrid, Spain

**Keywords:** Dental education, Rubric, Endodontics training, Portfolio evaluation, Evaluators´ agreement

## Abstract

**Background:**

Students´ assessment should be carried out in an effective and objective manner, which reduces the possibility of different evaluators giving different scores, thus influencing the qualification obtained and the consistency of education. The aim of the present study was to determine the agreement among four evaluators and compare the overall scores awarded when assessing portfolios of endodontic preclinical treatments performed by dental students by using an analytic rubric and a numeric rating scale.

**Methods:**

A random sample of 42 portfolios performed by fourth-year dental students at preclinical endodontic practices were blindly assessed by four evaluators using two different evaluation methods: an analytic rubric specifically designed and a numeric rating scale. Six categories were analyzed: radiographic assessment, access preparation, shaping procedure, obturation, content of the portfolio, and presentation of the portfolio. The maximum global score was 10 points. The overall scores obtained with both methods from each evaluator were compared by Student’s t, while agreement among evaluators was measured by Intraclass correlation coefficients (ICC). The influence of the difficulty of the endodontic treatment on the evaluators´ scores was analyzed by one-way ANOVA. Statistical tests were performed at a pre-set alpha of 0.05 using Stata 16.

**Results:**

Difficulty of canal treatment did not influence the scores of evaluators, irrespective of the evaluation method used. When the analytic rubric was used, inter-evaluator agreement was substantial for radiographic assessment, access preparation, shaping procedure, obturation, and overall scores. Inter-evaluator agreement ranged from moderate to fair with the numeric rating scale. Mean higher overall scores were achieved when numeric rating scale was used. Presentation and content of the portfolio showed slight and fair agreement, respectively, among evaluators, regardless the evaluation method applied.

**Conclusions:**

Assessment guided by an analytic rubric allowed evaluators to reach higher levels of agreement than those obtained when using a numeric rating scale. However, the rubric negatively affected overall scores.

## Background

European guidelines recommend that all dental school students should be competent in performing good quality root canal treatments upon graduation [1]. This is as part of a set of generic and subject-specific competences and abilities, essential to begin independent, unsupervised dental practice [2]. The provision of best possible dental treatment to the patients can only be achieved with the commencement of preceding preclinical courses and their success [3]. Specifically, students should gain adequate experience in the treatment of molar teeth in a preclinical environment [1]. This endodontic training should allow students to obtain fine psychomotor skills and to apply a previously-acquired robust academic knowledge [4].

The implementation of portfolios as an assessment technique in dental education gives the students the opportunity to demonstrate their capabilities to analyze and interpret prior learning. Moreover, it gives them the chance to show their problem-solving capabilities by applying critical thinking and self-directed learning [5, 6].

The evaluation of students’ performance on preclinical and clinical courses relies on the assessment of different members of the faculty. These assessments should be objective and reflective of both students’ knowledge and performance, looking for being consistent and standardized among all examiners [7]. An assessment procedure should provide validity, reliability, effectiveness and efficiency and its purpose should be clear to both, assessor and assessed [8]. In addition, it should provide immediate and comprehensive feedback to students on their performance so that they may learn from the experience [8]. In this sense, the consistency of the evaluator is crucial in the teaching and learning process, as it can affect students’ confidence and performance [9].

A rubric is a scoring tool for qualitative rating of authentic or complex student work scaled with levels of achievement and clearly defined criteria related to each level and placed in a grid [10, 11]. Two main categories of rubrics may be distinguished: holistic and analytical. In holistic scoring, the evaluator makes an overall judgment about the quality of performance, while in analytic scoring, the evaluator assigns a score to each of the dimensions being assessed in the task [11]. They have been found to be a promising reliable assessment element in dental education [12] as they provide a source of feedback to the students [13, 14] and the possibility to guide them to desired performance levels [12] whilst providing consistency in the evaluations among different examiners [3]. In fact, the unavoidable elements of subjectivity present in preclinical procedures might be reduced with the adoption of a grading rubric since it specifies teaching and learning outcomes for both teacher and student [10], while acceptable levels of inter-evaluator reliability can be achieved [15–17].

In Dentistry, rubrics have been used for the evaluation of students in different situations: oral presentations in Orthodontics [12] and Periodontics [15], preclinical training in Integrated Dentistry [13] and Prosthodontics [3], clinical performance in Periodontics [7] and for students’ self-assessment [13, 15, 18]. They have also been used to examine their reflective ability in e-portfolios [17]. However, information regarding the use of rubrics in the evaluation of endodontic treatments is scarce [13, 19].

Therefore, the aims of this study were to: (1) Determine the levels of agreement among four evaluators in the assessment of portfolios compiled by undergraduate dental students of endodontic preclinical treatments using an analytic rubric and a numeric rating scale, and (2) Compare the overall scores awarded to dental students after the evaluation of portfolios of endodontic preclinical treatments using both methods. Accordingly, the null hypotheses to be tested were: (1) Similar levels of agreement among different evaluators are found when using an analytical rubric and a numeric rating scale and, (2) The use of an analytical rubric results in similar overall scores to evaluation with a numeric rating scale.

## Materials and methods

### Preclinical endodontic treatments

The present investigation was carried out at Rey Juan Carlos University (Madrid, Spain) once the Ethics committee of this institution determined that its express permission was not necessary. Sixty-two undergraduate students performed root canal treatments in hand-held extracted human molars (six root canal treatments per student), to be prepared for their first endodontic treatments in patients. This training was part of preclinical practices in the subject of Dental Pathology and Restorative Dentistry II, during the fourth year of the degree and the second year in which the students worked in preclinical endodontics. This study was carried out after the assessment of the subject, so the students’ grades were not affected by their results. Teeth were supplied and selected by the students themselves, according to the following exclusion criteria: substantial loss of tooth structure, radiographically not visible canal paths, canal obliteration, extreme curvatures, incomplete root formation, extensive apical resorption, and internal resorption. Selection of molars was supervised by the teachers, who advised the students on possible anatomical aspects that could increase the complexity of the endodontic treatment. Once the teeth were selected, initial radiographs were taken. Using these diagnostic radiographs, the approximate working length (WL) of each root canal was measured. The access cavity was performed with high-speed diamond burs under refrigeration and the root canals were located using an endodontic probe. The students scouted root canals with K-file diameter 10, achieving apical patency at WL + 0.5 mm. Irrigation with 5.25% sodium hypochlorite delivered by syringe was kept throughout the entire shaping procedure. Students were asked to perform two treatments with hand files, one with continuous rotary motion (Protaper Next), one with reciprocating motion (Reciproc Blue), and other two treatments with a mechanized instrumentation of their choice. No intervention was made in the allocation of teeth according to the instrumentation technique. The instruments and techniques used for each treatment are shown in Table 1. Obturation technique was lateral condensation in all cases, using AH Plus sealer (Dentsply Sirona) and 0.02 standard gutta-percha points (Dentsply Sirona). For radiographic registration periapical size 2 EF-speed X-ray films (Henry Schein, Melville, NY, USA) were used. The X-ray generator used was a Kodak 2200 Intraoral X-ray System (Carestream Dental, Atlanta, GA, USA) operated at 65 kV-DC and 7 mA. Films were processed manually using Carestream Dental X-ray processing chemicals (Carestream Dental).

**Table 1:** Instruments used for each root canal treatment procedure

Instruments	Technique sequence
Hand K-files and K-flexible files	Step-back technique
Protaper Next (Dentsply Sirona)	Glide-path up to Hand file diameter 20 X1, X2 to WL Apical calibration with Hand file diameter 25 X3 (if needed)
Reciproc Blue (Dentsply Sirona)	Glide-path up to Hand file diameter 20 R25 to WL Apical calibration with Hand file diameter 25 R40 (if needed)

### Evaluation process of portfolios

Once the preclinical practices period was concluded, students compiled a digital descriptive portfolio for each of the six root canal treatments performed. These portfolios included: initial, WL, and obturation radiographs, photographs of the access cavity, step-by-step information about selected instruments, shaping procedure (manual, continuous rotation, or reciprocating motion) and obturation technique. They were also asked to describe the challenges faced during the whole process.

A random selection yielded 42 portfolios, representing 42 molars with root canal treatments to be evaluated by four evaluators. This minimum sample size was calculated accepting an alpha risk of 0.05 and a beta risk of 0.2 in a two-sided test, expecting to find an Intraclass correlation coefficients (ICC) of 0.7 or greater in the final ratings among evaluators. These evaluators were teachers in the subject of Dental Pathology and Restorative Dentistry II and postgraduate in Endodontics with more than ten years of clinical endodontics experience. However, they were not involved in the portfolios´ selection and kept blind as to the authorship of them. First, they jointly categorized the complexity of root canal anatomy of each molar, based on visual and radiographic inspection, and according to the case difficulty assessment form by the American Association of Endodontists (http://www.aae.org/caseassessment/). The molars were classified with the following difficulty: minimal (n = 10), moderate (n = 26), and high (n = 6). They also recorded the number of cases treated with each of the instrumentation techniques: hand K-files (n = 11), Protaper Next (n = 28), and Reciproc Blue (n = 3).

Afterwards, the 42 root canal treatments were individually evaluated by each examiner using two methods: an analytic rubric and, six months later, a numeric rating scale. The evaluators divided their analysis into 3 sessions for each evaluation method, on different days, evaluating 14 portfolios in each session (n = 42) and following the same order and with no evaluation time limit. Both methods were scored based on a ten-point scale that included six categories. These categories were weighted and distributed as follows: radiographic assessment (1 point), access cavity (2.5 points), shaping procedure (2.5 points), obturation (2.5 points), content of the portfolio (1 point) and presentation of the portfolio (0.5 point).

The analytic rubric resembled a grid with the categories listed in the leftmost column and five levels of performance (unsatisfactory, needs improvement, meets expectations, exceeds expectations and outstanding) distributed across the row with a corresponding pre-set score. This analytic rubric was specifically designed for the evaluation of the endodontic preclinical treatments and the calibration of its use among examiners was carried out prior to the evaluation of the portfolios. Details regarding the specific criteria and pre-set scores for each category can be accessed using the following DOI 10.21950/DPNC8Q.

Once all portfolios were assessed using both methods, points obtained from the six categories were added together to achieve an overall score between 0 and 10 that awarded the student a qualitative rating of: failed (0-4.9), approved (5-6.9), remarkable (7-8.9) or outstanding (9–10), as contemplated by Spanish Royal Decree 1125/2003 regulating the European credit and qualifications system in official university degrees [20].

### Statistical analysis

The influence of the degree of difficulty and the instrumentation technique on the evaluations by each teacher using both methods (rubric and numeric rating scale) were analyzed by one-way ANOVA test. Intraclass correlation coefficients (ICC) were used to test the agreement among the four evaluators for each category as well as for the overall scores obtained when the rubric and the numeric rating scale were used. Subsequently, overall scores obtained by the students with both methods of evaluation were also compared using Student´s t test and level of agreement with ICC. Individual measures were used in the ICC calculation process. Pass-fail and qualifications (failed, approved, remarkable, outstanding) agreements were calculated using Kappa index and quadratic weighted Kappa, respectively. Reliability results were categorized using the Landis and Koch criteria [21]: poor agreement (0), slight agreement (0.01–0.20), fair agreement (0.21–0.40), moderate agreement (between 0.41 and 0.60), substantial agreement (between 0.61 and 0.80) and almost perfect agreement (between 0.81 and 1.00). All statistical tests were performed at a pre-set alpha of 0.05 using Stata/IC 16.1 (Stata Corp LLC, College Station, TX, USA).

## Results

One-way ANOVA analysis showed that the ratings of each evaluator were not influenced by the difficulty of the treatment nor the instrumentation technique (p > 0.05), irrespective of the evaluation method used, and therefore, they were not considered in the subsequent analyses.

Descriptive results of the six categories and overall scores are shown in Table 2. When the rubric was used, inter-evaluator agreement among the four evaluators was substantial for categories associated with the root canal treatment, namely, radiographic assessment, access preparation, shaping procedure and obturation. On the other hand, when a numeric rating scale was used, inter-evaluator agreement was moderate for the same categories, except for shaping procedure, where agreement was fair. Presentation and content of the portfolio had slight and fair agreement with both methods of evaluation (Table 2). In overall scores, agreement was substantial with the rubric and moderate with a numeric rating scale (Table 2).

**Table 2:** Descriptive scores by category, overall score, and degree of inter- and intra-evaluator agreement using the two evaluation methods

Categories	Evaluation method	E1			E2			E3			E4			
		*Mean (SD)*	*Min*	*Max*	*Mean (SD)*	*Min*	*Max*	*Mean (SD)*	*Min*	*Max*	*Mean (SD)*	*Min*	*Max*	ICC*
**Radiographic assessment**	R	0.68 (0.27)	0.25	1.00	0.61 (0.28)	0	1.00	0.65 (0.26)	0.25	1.00	0.60 (0.23)	0.25	1.00	0.694
	NRS	0.52 (0.17)	0.20	0.80	0.48 (0.18)	0.10	0.80	0.66 (0.16)	0.30	1.00	0.62 (0.18)	0.20	0.90	0.450
**Access cavity**	R	1.27 (0.59)	0	2.50	1.27 (0.63)	0	2.50	1.38 (0.58)	0	2.50	1.24 (0.64)	0	2.5	0.604
	NRS	1.42 (0.39)	0.50	2.30	1.21 (0.42)	0.30	2.30	1.70 (0.49)	0.30	2.50	1.41 (0.41)	0.50	2.30	0.535
**Shaping procedure**	R	1.56 (0.92)	0	2.50	1.35 (0.56)	0.63	2.50	1.28 (0.60)	0	2.50	1.25 (0.58)	0.63	2.5	0.617
	NRS	1.68 (0.45)	0.80	2.30	1.35 (0.40)	0.50	2.00	1.79 (0.40)	0.50	2.30	1.75 (0.35)	1.30	2.30	0.310
**Obturation**	R	1.1 (0.70)	0	2.50	1.23 (0.72)	0	2.50	1.19 (0.61)	0	2.50	1.01 (0.57)	0	2.50	0.606
	NRS	1.43 (0.44)	0	2.30	1.42 (0.51)	0.30	2.30	1.71 (0.41)	0.30	2.30	1.85 (0.30)	1.00	2.30	0.536
**Presentation of the portfolio**	R	0.35 (0.14)	0	0.5	0.13 (0.16)	0	0.5	0.21 (0.13)	0	0.5	0.19 (0.12)	0	0.5	0.184
	NRS	0.39 (0.06)	0.30	0.50	0.32 (0.06)	0.20	0.40	0.37 (0.07)	0.20	0.50	0.36 (0.07)	0.20	0.50	0.300
**Content of the portfolio**	R	0.51 (0.29)	0	1.00	0.62 (0.24)	0	1.00	0.48 (0.22)	0	0.75	0.51 (0.25)	0	1.00	0.351
	NRS	0.71 (0.12)	0.50	1.00	0.59 (0.13)	0.30	0.80	0.69 (0.14)	0.30	0.90	0.66 (0.14)	0.30	0.90	0.346
**Overall score**	R	5.47 (1.97)	2	8.87	5.21 (1.77)	2.50	10.00	5.20 (1.63)	2.88	9.75	4.80 (1.63)	2.26	9.37	0.696
	NRS	6.05 (1.14)	4.40	8.20	5.27 (1.28)	2.80	7.80	6.85 (1.24)	4.40	9.10	6.59 (1.00)	4.70	8.90	0.502
ICC**		0.621			0.772			0.469			0.301			
**P**		0.01			NS			<0.01			<0.01			

SD: standard deviation; Min: minimum value; Max: maximum value; P: statistical significance between both methods of evaluation in overall scores (p <0.05); E1: evaluator 1; E2: evaluator 2; E3: evaluator 3; E4: evaluator 4; R: rubric; NRS: numeric rating scale; NS: not significant; ICC* of scores among 4 evaluators´ independent assessment for each method of evaluation (analytic rubric vs. numeric rating scale) in each category and overall scores. ICC** between both methods (analytic rubric vs. numeric rating scale) in overall scores for each evaluator (p<0.001)

**Fig. 1:**
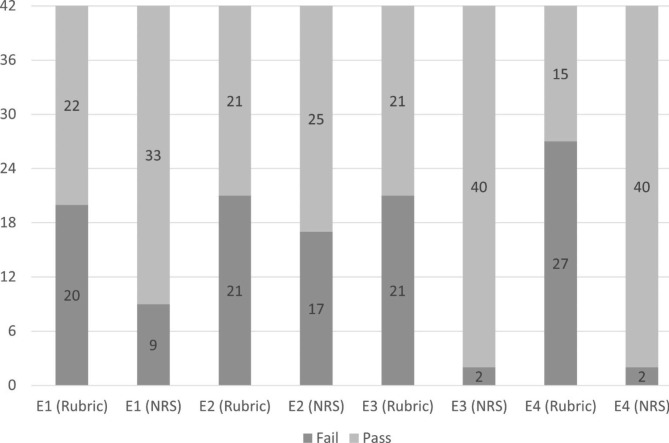
Distribution of fail-pass scores given by the evaluators (E1, E2, E3, E4) (n = 42) using a rubric (R) and a numeric rating scale (NRS)

Pass-fail distribution of overall portfolio scores for all possible pairs of evaluators is shown in Fig. 1, while Table 3 shows pass-fail agreement results by the evaluators. When the rubric was used, agreement was moderate in all cases, except for E1-E4 where agreement was fair. In contrast, when the numeric rating scale was used, agreement was moderate just for one pair (E3-E4) whilst for the remaining pairs agreement was lower, including three pairs with slight agreement.

**Table 3:** Agreement indexes in overall scores for all possible pairs of evaluators. Pass-fail; Qualifications (failed, approved, remarkable, outstanding); Overall numeric scores

	Pass-fail agreement*			Qualifications agreement**			Overall numeric scores agreement***	
Evaluators	R	NRS		R	NRS		R	NRS
E1-E2	0.571	0.358		0.712	0.485		0.679	0.630
E1-E3	0.571	0.309		0.700	0.538		0.722	0.601
E1-E4	0.390	0.112		0.444	0.477		0.563	0.569
E2-E3	0.619	0.137		0.777	0.291		0.816	0.393
E2-E4	0.523	0.021		0.690	0.376		0.657	0.397
E3-E4	0.428	0.475		0.666	0.401		0.768	0.541

R rubric; NRS numeric rating scale; * Kappa value; ** Quadratic weighted Kappa value (ordinal scales); *** Intraclass coefficient correlation (agreement and individual measures)

**Fig. 2:**
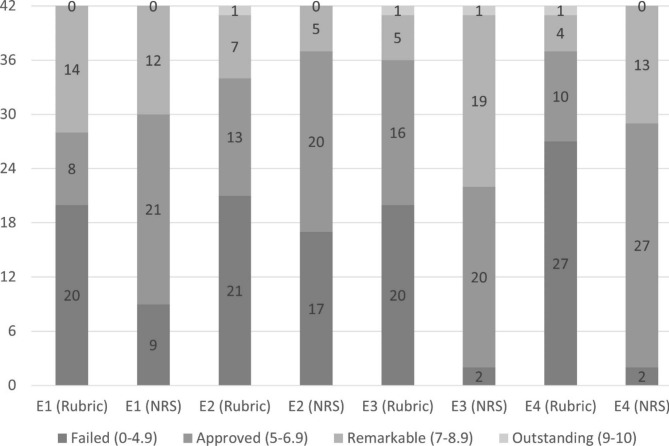
Distribution of qualifications (failed, approved, remarkable, outstanding) given by the evaluators (E1, E2, E3, E4) (n = 42) using a rubric (R) and a numeric rating scale (NRS)

Qualification distribution by the evaluators is shown in Fig. 2. Agreement among qualifications (failed, approved, remarkable, outstanding) was substantial in all pairs (except E1-E4) with the rubric. On the contrary, the numeric rating scale yielded only moderate and fair agreements (Table 3). With the use of the rubric, agreement in numeric scores was almost perfect between E2 and E3, moderate between E1 and E4, and substantial for the remaining pairs of evaluators. However, when a numeric rating scale was used, coefficients ranged from 0.393 to 0.630, being fundamentally fair and substantial (Table 3).

Regarding reliability between both methods in overall scores (analytic rubric vs. numeric rating scale) for each evaluator, agreement was substantial for E1 and E2, moderate for E3 and fair for E4 (p < 0.001) (Table 2). When the evaluations between both methods were compared, Student’s t test showed that with the use of a rubric mean overall scores were lower for E1, E3 and E4 (p < 0.05), while for E2 differences were not found (p > 0.05) (Table 2).

## Discussion

Higher levels of agreement among different evaluators were achieved when the rubric was used for five of the six categories tested and for overall scores, therefore, the first hypothesis must be rejected. Lower inter-evaluator agreement was detected in our study with the numeric rating scale, something that had been previously reported both by Jenkins et al. [22], using a global evaluation method, and AlHumaid et al. [23], using a rating scale which did not include descriptions of the levels of performance. According to Brennan [24], inter-evaluator reliability tends to be higher when tasks are standardized and scoring procedures are well defined.

However, Sharaf et al. [9] found no improvement in inter-evaluator agreement using analytical evaluation methods. They evaluated operative procedures performed by dental students in preclinical sessions and compared variability using two evaluation methods: glance and grade (global), and checklist and criteria (analytical) and reported a similar pattern of disagreement among evaluators.

Nevertheless, comparing our results with the studies mentioned above is not possible, as their methodology varied significantly. Procedures assessed ranged from dental preparations suitable for restorations [9, 22] to several specialties in the same study [23], and rubrics were not implemented in the evaluation process.

Preclinical dental training demands a low student-teacher ratio; thus, several teachers oversee students´ performance in the same academic course. In this sense, the rubric can be a valuable tool, because students’ scores are less dependent on the assigned teacher, and more on the specifications of the rubric. However, we expected to achieve even higher levels of agreement among the evaluators in all categories and overall score using the rubric. Noticeably, better levels of agreement were found in the most technical aspects of the root canal treatment (e.g., radiographic assessment, access cavity, shaping procedure and obturation) as well as in overall score, while the presentation and content of the portfolio failed to reach a consensus among the evaluators, even with the adoption of a rubric.

In our study, all the steps of the endodontic treatment were evaluated, in accordance with Vantorre et al. [25]. Root canal treatments are step-by-step interdependent, so it is reasonable to evaluate each step individually rather than to just evaluate the final result. Regarding the portfolio assessment, reflection and reflective writing are considered difficult skills [17]. The lower levels of inter-evaluator agreement found in presentation and content of the portfolio might be attributed to the fact that difficulty of tasks affects the level of agreement among evaluators [13, 26, 27]. Nonetheless, it is worth noting that when the ten-point scale was weighted and distributed among the six categories, these two were assigned lower values than the categories associated directly with the endodontic treatment, aiming for the overall scores to reflect more accurately the students’ practical skills.

Rubrics have been implemented in other dental faculties to assess students’ competence in preclinical endodontics, although categories and design of the rubrics varied among the consulted publications, the number of the adjacent achievement levels was either three [13, 19] or five [13]. Consensus agreement of evaluators strongly depends on the number of levels in the rubric, with fewer levels, there will be a greater chance of agreement [11, 13]. The fact that our rubric included five levels of achievement for each category gave us the opportunity to discriminate further from one adjacent achievement level to the next. However, this number of achievement levels might have hampered inter-evaluator agreement.

In many preclinical endodontic trainings artificial resin teeth are frequently used because they provide a standardized alternative [28–31], although they lack the ability to accurately reproduce dentin hardness [29–31]. For this reason, resin teeth were not considered suitable for students to become acquainted with root canal complex anatomy and the sensations of natural dental tissues. However, precisely because of the great morphological variability of these teeth, we had to ensure that the perception of difficulty did not influence the evaluators’ judgement, which was established at the outset.

The increased objectivity acquired with the use of a rubric was also evident when individual evaluations were subjected to paired test for three parameters (pass/fail, qualifications, and numeric scores) as a higher agreement could be observed for most pairs of evaluators (Table 2). Nevertheless, despite the improvement in agreement from the use of a numeric rating scale to the use of a rubric, from the students´ point of view, what matters most is the final numeric score and whether they pass or fail the evaluation. Therefore, the subjectivity that is still present, even with the use of a rubric, should also be addressed.

It should be highlighted that when the evaluations between both methods were compared, mean overall scores were lower with the use of a rubric (differences were found for three of the four evaluators), inferring that the use of an analytic rubric negatively affects students’ overall score. Therefore, the second hypothesis must be rejected. Moreover, when the rubric was used, the number of students that failed was particularly higher. This finding could be due to the fact that rubric is a more demanding assessment method, which highly compartmentalizes the qualifications and leads to more severe penalties when errors arise.

However, with the adoption of the rubric, all the evaluators scored the highest and the lowest values in most categories on some occasion. On the contrary, with the numeric rating scale, there were categories where none of them assigned the minimum nor the maximum score, for instance, access cavity, shaping procedure and presentation of the portfolio. The explanation might lie in the fact that numeric rating scales lack strictly defined performance standards.

The authors consider that a valuable element that the rubric provided, apart from already mentioned standardization, is the possibility of detailed and immediate feedback to the students, thus becoming a very practical and agile teaching instrument. This feedback effect might be seen when, in the same academic period, a student gradually performs endodontic treatments with higher scores. However, this could not be addressed in this study, as the sample was randomly selected.

Furthermore, students’ self-assessment through a rubric could improve their awareness of where their numeric grade lies and how to improve it. In fact, the use of rubrics as a useful self-assessment tool has been previously recommended [13, 15, 18]. Even though this was not registered in the present study, future studies using the rubric proposed by the authors could consider including students´ self-assessment as well.

## Conclusions

The use of an analytic rubric allowed different evaluators to reach higher levels of agreement than those obtained with a numeric rating scale in the evaluation of portfolios of endodontic treatments performed in a preclinical environment. Among the six categories that were evaluated, the two least related to root canal treatment and most associated with the portfolio itself (content and presentation of the portfolio), showed the lowest agreement among the evaluators, regardless of the method of evaluation applied.

The implementation of a rubric, on the other hand, negatively affected the students’ overall portfolio score.

## Data Availability

The datasets used and/or analyzed during the current study are available from the corresponding author upon reasonable request.
